# Retraction: “folic acid supplementation, dietary folate intake during pregnancy and risk for spontaneous preterm delivery: a prospective observational cohort study”

**DOI:** 10.1186/1471-2393-14-202

**Published:** 2014-07-03

**Authors:** Verena Sengpiel, Jonas Bacelis, Ronny Myhre, Solveig Myking, Aase Devold Pay, Margaretha Haugen, Anne-Lise Brantsæter, Helle Margrete Meltzer, Roy M Nilsen, Per Magnus, Stein Emil Vollset, Staffan Nilsson, Bo Jacobsson

**Affiliations:** 1 Department of Obstetrics and Gynaecology, Sahlgrenska Academy, Sahlgrenska University Hospital/Östra, SE-416 85 Göteborg, Sweden; 2 Department of Genes and Environment, Division of Epidemiology, Norwegian Institute of Public Health, P.O. Box 4404, Nydalen NO-0403 Oslo, Norway; 3 Department of Obstetrics, Oslo University Hospital, P.O. Box 4950, Nydalen NO-0424 Oslo, Norway; 4 Department of Exposure and Risk Assessment, Division of Environmental Medicine, Norwegian Institute of Public Health, P.O. Box 4404, Nydalen NO-0403 Oslo, Norway; 5 Department of Public Health and Primary Health Care, University of Bergen, NO-5018 Bergen, Norway; 6 Division of Epidemiology, Norwegian Institute of Public Health, P.O. Box 4404NO-0403 Oslo, Norway; 7 Norwegian Institute of Public Health and University of Bergen, Kalfarveien 31, NO-5018 Bergen, Norway; 8 Mathematical Sciences, Chalmers University of Technology, SE-412 96 Göteborg, Sweden

## Retraction

This article [1] has been retracted by the authors, as some of the reported data was incorrect. The error concerned approximately 8% of the cohort (women who answered the first version of the food frequency questionnaire, before 2002) and was caused by misclassification of the amount of the women’s reported folic acid supplementation (no supplement use instead of missing information on supplement use). The error led to minor changes in the results.

However, the estimate of the association between folate intake and spontaneous preterm delivery did not change the conclusions after the error was corrected.

In consultation with the Journal’s editors we decided to retract the article from BMC Pregnancy and Childbirth.

A corrected manuscript will be resubmitted to the Journal at the time of retraction.

On behalf of all authors,

Verena Sengpiel and Bo Jacobsson.

## Pre-publication history

The pre-publication history for this paper can be accessed here:

http://www.biomedcentral.com/1471-2393/14/202/prepub
